# Discovery of Retro-1 Analogs Exhibiting Enhanced Anti-vaccinia Virus Activity

**DOI:** 10.3389/fmicb.2020.00603

**Published:** 2020-04-23

**Authors:** Lalita Priyamvada, Philip Alabi, Andres Leon, Amrita Kumar, Suryaprakash Sambhara, Victoria A. Olson, Jason K. Sello, Panayampalli S. Satheshkumar

**Affiliations:** ^1^Poxvirus and Rabies Branch, Centers for Disease Control and Prevention, Atlanta, GA, United States; ^2^Department of Chemistry, Brown University, Providence, RI, United States; ^3^Immunology and Pathogenesis Branch, Influenza Division, Centers for Disease Control and Prevention, Atlanta, GA, United States

**Keywords:** viral inhibitor, poxvirus, antiviral agent, retrograde transport, vaccinia virus, Retro-1, retrograde inhibitor, ST-246

## Abstract

Orthopoxviruses (OPXVs) are an increasing threat to human health due to the growing population of OPXV-naive individuals after the discontinuation of routine smallpox vaccination. Antiviral drugs that are effective as postexposure treatments against variola virus (the causative agent of smallpox) or other OPXVs are critical in the event of an OPXV outbreak or exposure. The only US Food and Drug Administration-approved drug to treat smallpox, Tecovirimat (ST-246), exerts its antiviral effect by inhibiting extracellular virus (EV) formation, thereby preventing cell–cell and long-distance spread. We and others have previously demonstrated that host Golgi-associated retrograde proteins play an important role in monkeypox virus (MPXV) and vaccinia virus (VACV) EV formation. Inhibition of the retrograde pathway by small molecules such as Retro-2 has been shown to decrease VACV infection *in vitro* and to a lesser extent *in vivo*. To identify more potent inhibitors of the retrograde pathway, we screened a large panel of compounds containing a benzodiazepine scaffold like that of Retro-1, against VACV infection. We found that a subset of these compounds displayed better anti-VACV activity, causing a reduction in EV particle formation and viral spread compared to Retro-1. PA104 emerged as the most potent analog, inhibiting 90% viral spread at 1.3 μM with a high selectivity index. In addition, PA104 strongly inhibited two distinct ST-246-resistant viruses, demonstrating its potential benefit for use in combination therapy with ST-246. These data and further characterizations of the specific protein targets and *in vivo* efficacy of PA104 may have important implications for the design of effective antivirals against OPXV.

## Introduction

The *Orthopoxvirus* (OPXV) genus contains several human pathogens of public health concern, including variola virus (the causative agent of smallpox), monkeypox virus (MPXV), cowpox virus, and vaccinia virus (VACV, the virus that formulates the smallpox vaccine) (Fenner and Nakano, 1988). While smallpox has been eradicated, other OPXVs continue to pose a threat to human health due to the lack of protective immunity post the cessation of routine smallpox vaccination (Durski et al., 2018). In particular, the incidence of MPXV has been on the rise due to several recent outbreaks in West and Central Africa after a long hiatus in reported epidemics (Durski et al., 2018). There is also a risk of MPXV infections spreading outside the endemic region through travel or the importation of infected animals, as seen in several recent cases (Reed et al., 2004; Vaughan et al., 2018; Angelo et al., 2019; Erez et al., 2019). In addition, the identification of several new OPXVs over the past decade demonstrates the ongoing circulation and maintenance of these viruses in animal reservoirs, and highlights the need for surveillance and development of better diagnostic assays and therapeutics (Hoffmann et al., 2015; Vora et al., 2015; Springer et al., 2017; Lanave et al., 2018).

OPXVs are large double-stranded DNA viruses that have a complex life cycle, replicate in the cytoplasm of infected cells, and generate two distinct virion forms: mature virus (MV) and extracellular virus (EV), differentiated based on the number of membranes surrounding the central DNA core (Condit et al., 2006). Mature viruses consist of a viral core and a single viral membrane and harbor all the necessary proteins required for virus binding and entry. Although infectious, MVs remain inside an infected cell until lysis (Smith et al., 2002). A proportion of MVs undergo double-membrane wrapping to generate wrapped virus (WV), which can stay attached to the infected cell (cell-associated virus) or exit the cell (EV) (Smith et al., 2002; Moss, 2006). Although EV particles constitute only 1–10% of MVs, they are essential for cell-to-cell and long-distance viral spread and therefore play an important role in OPXV pathogenesis (Payne, 1980; Blasco and Moss, 1992; Engelstad and Smith, 1993). VACV mutants that fail to generate EVs produce small plaques *in vitro* and are attenuated *in vivo*, even in severely immunodeficient animal models (Blasco and Moss, 1991; Engelstad and Smith, 1993; Wolffe et al., 1993; Grosenbach et al., 2010).

The importance of EV formation for VACV pathogenesis is further highlighted by the fact that pharmacological intervention is efficacious in suppressing infection. At present, tecovirimat (ST-246) is the only US Food and Drug Administration-approved antiviral available for postexposure treatment against smallpox. ST-246 targets the viral envelope protein F13 required for EV formation, thereby decreasing viral spread and infection-induced pathology (Yang et al., 2005; Grosenbach et al., 2011). However, prolonged treatment of ST-246 can lead to the generation of drug-resistant viruses due to mutations in the F13L gene, as documented in a progressive vaccinia (PV) patient in 2009 (Lederman et al., 2012). A single amino acid mutation in F13L may overcome the therapeutic effect of the drug, reinforcing the need for additional therapeutic candidates that are safe and effective against OPXVs.

In our efforts to develop new therapeutic strategies, we focused on the peculiar dependence of OPXVs on the retrograde transport pathway for EV formation. While several viruses (Simian virus 40, papillomavirus, and influenza) rely on retrograde transport for entry into a cell, OPXVs require retrograde transport for EV membrane wrapping, and consequently viral egress (Spooner et al., 2006; Harrison et al., 2016; Sivan et al., 2016). We and others have previously demonstrated that impeding the retrograde pathway by targeting the Golgi-associated retrograde protein complex can greatly reduce EV yield with minimal impact on MV formation in VACV and MPXV (Harrison et al., 2016; Realegeno et al., 2017). Treatment with small molecule inhibitors of the retrograde pathway such as Retro-1 and Retro-2 significantly decreased EV formation *in vitro* by preventing the trafficking of the viral protein F13, which is required for membrane wrapping (Harrison et al., 2016; Sivan et al., 2016). However, initial characterizations of the antiviral efficacy of Retro-2 *in vivo* yielded underwhelming results, with treated mice showing marginal improvement in signs of clinical disease and comparable lung viral titers compared to untreated mice (Harrison et al., 2016). Additionally, better clinical scores were only observed in animals pretreated with Retro-2, and not in mice treated only postinfection (Harrison et al., 2016). Given the highly selective, potent anti-VACV activities of Retro-1 and Retro-2 *in vitro*, we were interested in structurally optimizing these small molecule inhibitors to identify novel antivirals with greater inhibitory effect compared to the parent compounds *in vitro*, as well as better *in vivo* therapeutic efficacy.

To this end, we screened a diverse collection of greater than 80 compounds sharing a benzodiazepine scaffold like Retro-1 for efficacy in *in vitro* anti-VACV assays. These efforts led to the identification of a potent antiviral we have named PA104. Like Retro-1 and Retro-2, PA104 can potently inhibit EV formation and VACV spread, while minimally impacting MV yield and causing little to no cytotoxicity. We also show that the reduction in viral spread by PA104 is attributable to inhibition of EV formation as evidenced by lack of virus secreted in media and impaired actin tail formation in infected cells. It is particularly noteworthy that PA104 also inhibits viral spread of two distinct ST-246-resistant viruses, establishing its potential for use in postexposure treatment in combination with ST-246. These observations warrant further investigation of PA104 and its efficacy as an antiviral agent.

## Materials and Methods

### Viruses, Cell Lines, and Inhibitors

Vaccinia virus WR-GFP, VACV WR A4-YFP, VACV WR-Luc, and VACV IHDJ stocks were grown in BSC40 cells with Dulbecco modified eagle medium (DMEM) containing 2% fetal bovine serum (FBS). Two ST-246-resistant viruses were used in this study, each containing distinct mutations in the F13L gene: the first (A290V) was isolated from a 2009 human PV case (Lederman et al., 2012) and thereafter passaged in our laboratory, and the second (N267D) was provided by SIGA Technologies (Smith et al., 2011). These two viruses will hereby be referred to as RV1 and RV2, respectively. All viral stocks were titered by plaque assay prior to use in experiments. BSC40 and HeLa cell lines were passaged in DMEM with 10% FBS and 10 units/mL of penicillin and 100 μg/mL of streptomycin (penicillin–streptomycin). For all experiments involving viral infection, DMEM containing 2% FBS, hereby referred to as “DMEM-2,” was used as a diluent or culture media.

The novel compounds in this study were synthesized in-house using diversity-oriented synthetic approaches. Our synthetic approach involved two different six- and four-step synthetic schemes that were utilized (based on synthetic limitations: cost, the scope of reactions, readily available starting materials) at different stages of the SAR. The specific retrosynthetic disconnections that were effected to access these molecules are shown below. The exact details of these reaction modules/improvisations and navigations of synthetic constraints will be presented in a subsequent publication. Meanwhile, additional information about the compounds can be made available upon request.


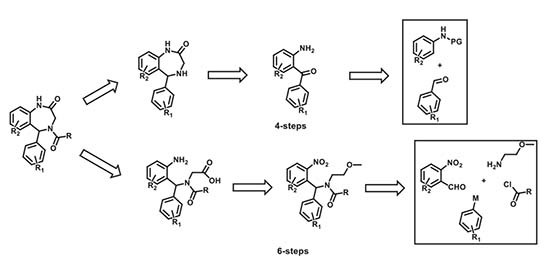


### Viral Spread Assay

The viral spread assay was performed as previously described (Cryer et al., 2017). A serial dilution series of retrograde inhibitor compounds was mixed 1:1 (vol/vol) with VACV-WR-GFP virus diluted in DMEM-2 at multiplicity of infection (MOI) 0.3. The mixture was transferred to black 96-well, clear-bottom plates (06-443-2; Corning, NY, United States) containing HeLa cells. Each plate included four to eight wells of the following controls: uninfected cells, no treatment (virus only), and virus + AraC (cytosine arabinoside, 40 μg/mL), and each dilution series was tested in duplicate. The cells were infected with the virus + compound mixture at 37°C for 24 h. Following infection, the cells were washed once with phosphate-buffered saline (PBS) and fixed with 4% paraformaldehyde for 15 min at room temperature (RT). Cells were then washed with PBS and stained with 4’,6-diamidino-2-phenylindole (DAPI) for nuclei visualization for 10 min at RT. The plates were imaged using the ArrayScan XTI High Content Screen (HCS) reader, and the percentage of GFP and DAPI positive cells was quantified using the HCS Studio Cell Analysis software as previously described (Johnson et al., 2008). Total viral spread and viral spread for each compound were measured as shown below:

$$Totalviralspread=\%GFP_{v\,i\,r\,u\,s\,o\,n\,l\,y}^{+}-\%GFP_{A\,r\,a\,C}^{+}$$

$$V\,i\,r\,a\,l\,s\,p\,r\,e\,a\,d\,f\,o\,r\,c\,o\,m\,p\,o\,u\,n\,d=\frac{\%GFP_{C\,o\,m\,p\,o\,u\,n\,d}^{+}-\%GFP_{A\,r\,a\,C}^{+}}{T\,o\,t\,a\,l\,v\,i\,r\,a\,l\,s\,p\,r\,e\,a\,d}$$

Viral spread data were analyzed using a non-linear regression (curve fit) with the sigmoidal dose–response variable slope equation to determine the concentrations required for 50% inhibition (IC_50_) and 90% inhibition (IC_90_) of viral spread relative to the no treatment control. Analyses were performed using GraphPad Prism software (GraphPad Software, v7, San Diego, CA, United States). Values were determined based on two or more replicates from two independent experiments.

### Cytotoxicity Assay

The potential cytotoxic effects of the retrograde inhibitor compounds were tested at a range of concentrations spanning 400–0.3 μM. HeLa cells seeded in 96-well plates were infected with a mixture of VACV WR (MOI 0.01) and serially diluted compounds at 37°C for 24 h. Each concentration was tested in duplicate. Supernatants were collected, and the levels of extracellular lactate dehydrogenase (LDH) released in the supernatants were quantified using the LDH Cytotoxicity Assay Kit (88953; Thermo Scientific Pierce, Waltham, MA, United States) as per the manufacturer’s instructions. Cytotoxicity data were analyzed using a non-linear regression (curve fit) with the sigmoidal dose–response variable slope equation to determine the concentrations required for 50% LDH signal (CC_50_) relative to the positive control (lysed cells). This analysis was performed using GraphPad Prism software (GraphPad Software, v7). Values were determined based on two or more replicates from two independent experiments.

### Entry Assay

BSC40 cells were infected with VACV WR-Luc virus at MOI 3 for 1 h at RT. After infection, cells were washed three times with PBS to remove unbound virus. Retrograde inhibitor compounds diluted to 2.5 μM in DMEM-2 were added to cells and incubated at 37°C for 2 h. All compounds were tested in duplicate. Reporter lysis buffer was added to the wells to lyse cells, and luciferase activity was measured using the Luciferase Assay System (Promega, Madison, WI, United States) according to manufacturer’s instructions. Luciferase activity was measured using an ENSPIRE plate reader (PerkinElmer, Waltham, MA, United States).

### MV and EV Quantification

BSC40 cells were infected with VACV IHDJ virus at MOI 3 for 1 h at RT. The infected cells were washed three times with DMEM-2 to remove excess virus, and retrograde inhibitor compounds were then added to the cells at a concentration of 2.5 μM. Each compound was tested in triplicate, and the cells were incubated with the compounds for 24 h at 37°C. Supernatants were collected and frozen down to determine EV yield. To measure MV yield, cells were washed three times with PBS to remove any EV particles, collected using a cell scraper, and freeze-thawed three times to release MV particles. Extracellular virus and MV yield was quantified by titering the supernatants and cells, respectively, by plaque assay. The percent EV formation and EV inhibition were determined as follows:

$$\%\,E\,V\,f\,o\,r\,m\,a\,t\,i\,o\,n=\frac{E\,V\,y\,i\,e\,l\,d}{\left(E\,V\,y\,i\,e\,l\,d+M\,V\,y\,i\,e\,l\,d\right)}$$

$$\%\,E\,V\,I\,n\,h\,i\,b\,i\,t\,i\,o\,n\,f\,o\,r\,c\,o\,m\,p\,o\,u\,n\,d=100-\frac{\%EVformation_{C\,o\,m\,p\,o\,u\,n\,d}}{\%EVformation_{V\,i\,r\,u\,s\,o\,n\,l\,y}}$$

Statistical significance was measured using a one-way analysis of variance (ANOVA) test. MV end EV yields in the presence of the retrograde inhibitor compounds and ST-246 were compared to the virus only control using *post hoc* Sidak multiple-comparisons test.

### Effect on ST-246-Resistant Viruses

BSC40 cells seeded in 12-well plates were infected with approximately 100 plaque forming units (pfu) of VACV WR, RV1, or RV2 for 1 h at 37°C. The virus was removed and compounds PA24, PA63, and PA104 were added to the cells at a concentration of 10 μM, and ST-246 at 2 μM diluted in DMEM containing 2% carboxymethylcellulose. After a 72-h incubation with the inhibitors at 37°C, the cells were stained with crystal violet (CV) containing 4% paraformaldehyde for 15 min at RT. Post-CV staining, the plates were washed with water and imaged.

### Confocal Imaging

BSC40 cells were seeded in 24-well glass- bottom tissue culture plates (MatTek Corporation, Ashland, MA, United States) and infected with VACV WR A4-YFP (MOI 1) for 1 h at 37°C. The virus was removed, and compounds PA24, PA63, and PA104 were added to the cells at a concentration of 10 μM. After a 24-h incubation with the inhibitors at 37°C, cells were washed with PBS and fixed with 4% paraformaldehyde for 15 min at RT. Cells were stained with Alexa Flour 594 Phalloidin (ThermoFisher, Waltham, MA, United States) for actin visualization and with DAPI for nuclei visualization. After staining, cells were mounted with Prolong Gold Antifade Mounting media (Molecular Probes, Eugene, OR, United States) and imaged using the LSM 710 inverted confocal microscope (Zeiss, Oberkochen, Germany). Virus-loaded actin tails in infected cells were counted for each condition (number of cells counted per condition ranging between 5 and 9) and compared for statistical significance using a one-way ANOVA test.

### Isolation of ST246-Resistant Virus From PV Case

BSC40 cells were infected with serially diluted culture material isolated from a 2009 PV patient (Lederman et al., 2012) in the presence or absence of ST-246. Plaques observed in the presence of ST-246 were picked and reconstituted in DMEM-2. Plaque purification was performed by serially passaging single plaques grown in the presence of ST-246 four times. The isolated virus was grown in a six-well plate of BSC40 cells to create an RV1 viral stock. To confirm the presence of the previously characterized A290V mutation in RV1, the F13L gene was sequenced by Sanger sequencing.

## Results

### Screen of Benzodiazepines Yields Inhibitors of VACV Spread *in vitro*

**FIGURE 1:**
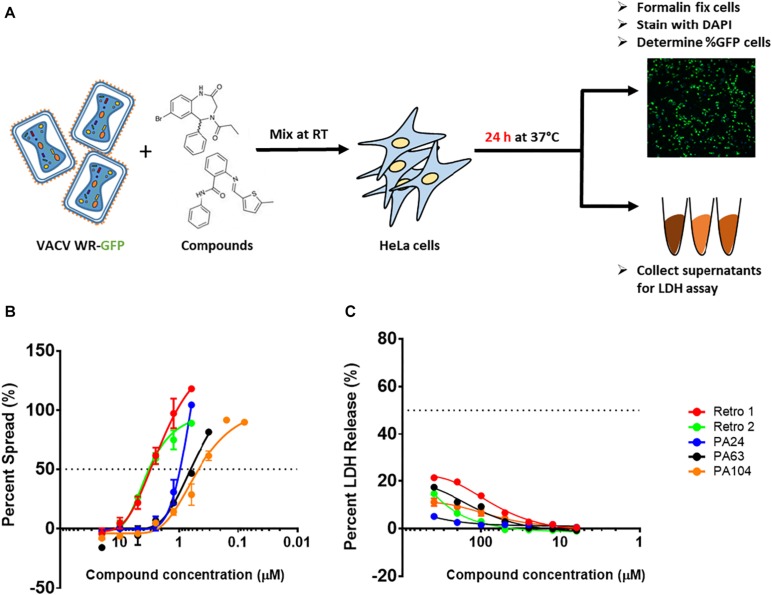
Structural analogs of Retro-1 can inhibit VACV spread with minimal cytotoxicity. **(A)** Schematic representation of steps used to determine viral spread and cellular toxicity in the presence of Retro-1 analogs. The virus spread and cytotoxicity assays were performed twice for each compound, in duplicate. **(B)** Representative graph showing percent viral spread in the presence of a serial dilution series of five retrograde inhibitors. Values plotted represent the average of two independent experiments. **(C)** Representative graph showing percent LDH release in the presence of a serial dilution series of five retrograde inhibitors. Values plotted represent the average ± SEM from two independent experiments.

To identify new inhibitors of VACV infection, we quantified viral spread in the presence of diverse compounds sharing a core structure with Retro-1 using VACV WR-GFP as shown in Figure 1A. A total of 83 compounds were tested, and the percentage of GFP^+^ cells was determined by fluorescent imaging. We measured viral spread for each compound over a range of concentrations (Figure 1B) and determined the 50% inhibitory concentrations (IC_50_) for all and the 90% inhibitory concentrations (IC_90_) for a subset of the compounds. Of the compounds tested, 52 showed > 50% inhibition of VACV spread at concentrations ≤ 10 μM. Within this subset, 17 compounds showed greater inhibitory potency than Retro-1 and Retro-2 and had IC_50_ and IC_90_ values ranging between 0.5 and 2.0 μM and between 1.3 and 5.2 μM, respectively (Table 1, data not shown). In addition to examining their effect on viral spread, we also assessed the cellular cytotoxicity of a subset of compounds by measuring cell-released LDH levels (Figure 1C). We observed that a majority of potently inhibitory compounds exhibited low cytotoxicity even at 400 μM. Based on the viral spread screen of the Retro-1 like benzodiazepines, we narrowed our focus to three of the most potent VACV inhibitors: PA24, PA63, and PA104 (Table 1).

**TABLE 1:** Inhibitory and cytotoxic activities of select compounds.

**Compound**	**IC50 (μM)**	**IC90 (μM)**	**CC50 (μM)**	**SI**
ST-246	0.04	0.1	>400	>10000
Retro 1	3.0	7.2	>400	>135
Retro 2	2.2	5.5	>400	>194
PA104	0.5	1.3	>400	>800
PA63	0.7	1.7	>400	>570
PA24	1.0	1.8	>400	>400

### Retro-1 Derivatives Inhibit VACV Infection Post-entry

**FIGURE 2:**
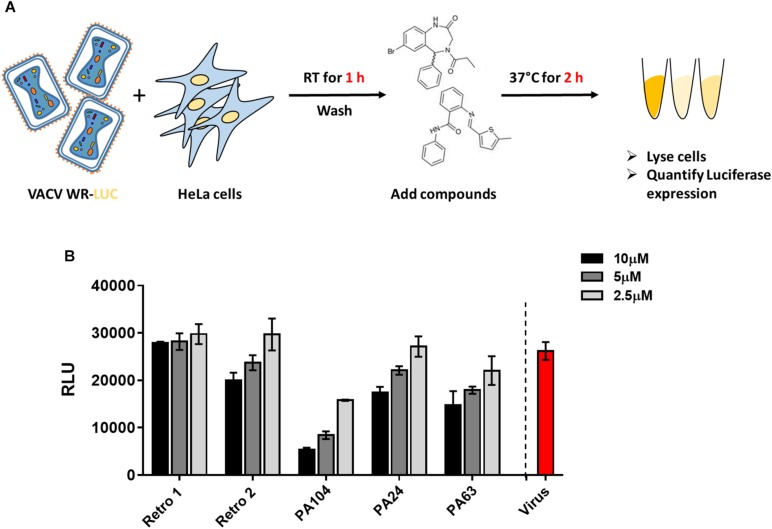
Effect of Retro-1 analogs on early stages of VACV infection. **(A)** Schematic representation of VACV entry assay. **(B)** Relative light units (RLUs) in the presence of select retrograde inhibitors diluted to 2.5 μM (light gray), 5 μM (dark gray), and 10 μM (black). Values plotted represent the average of two independent experiments.

Given the considerable reduction in viral spread described earlier, we checked the effect of PA24, PA63, and PA104 on VACV entry. We infected cells with VACV WR-Luc at RT, which allowed virus attachment but not entry, washed cells to remove unbound virus, and subsequently added the inhibitors (Figure 2A). After a 2-h incubation at 37°C, we determined luciferase expression, controlled by a synthetic early/late promoter, as a surrogate for early protein synthesis. As shown in Supplementary Figure S1A, input luciferase activity was very low, suggesting that the RLU values in our assay were largely attributable to viral entry and gene expression, rather than prepackaged luciferase.

We observed that PA24 and PA63 did not markedly decrease viral entry at the lower concentrations tested, corroborating the findings of a previous study that tested Retro-1 and Retro-2 (Sivan et al., 2016) (Figure 2B). While a higher concentration (10 μM) of PA24 and PA63 reduced viral entry, for PA104 a decrease in viral entry was observed at all three concentrations tested (Figure 2B). To exclude the possibility that these compounds affect the enzymatic activity of luciferase, we added different concentrations of PA104 to lysed VACV WR-Luc-infected cells just before the addition of the substrate. We found no difference in luciferase activity between the treatment groups (Supplementary Figure S1B). Together, these data demonstrate that potent retrograde inhibitors may inhibit virus entry at high concentrations.

### Reduced EV Yield in the Presence of Retro-1 Derivatives

**FIGURE 3:**
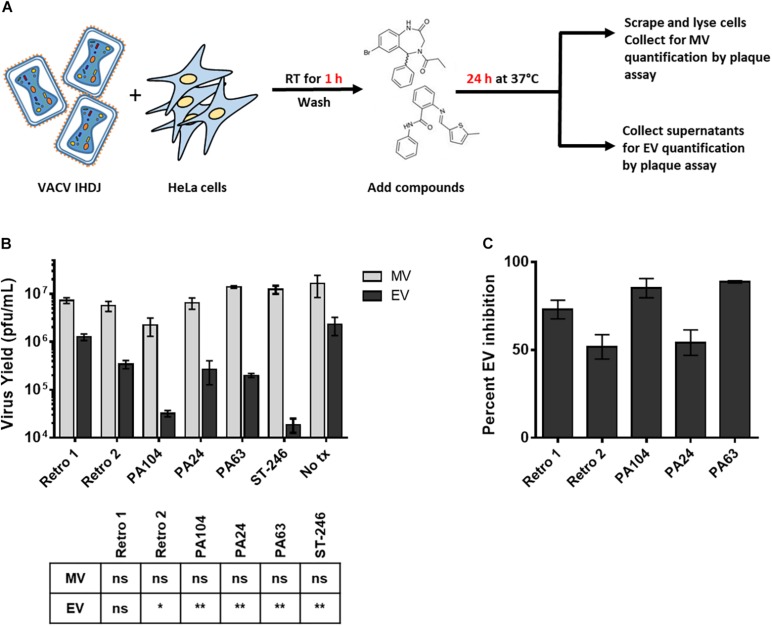
Reduced EV formation in the presence of Retro-1 analogs. **(A)** Schematic representation of assay used to determine MV and EV yield in the presence of Retro-2 and Retro-1 analogs. **(B)** Mature virus (light gray) and EV (black) yield (pfu/mL) in the presence of 2.5 μM retrograde inhibitors. Values represent the average of three replicates. The results of one-way analysis of variance and *post hoc* Sidak multiple-comparisons test comparing the three replicates (*n* = 3) of each compound to no treatment control are tabulated below graph: ns = not significant, *p ≤ 0.05 and **p ≤ 0.005. **(C)** Percent inhibition of EV formation in the presence of 2.5 μM retrograde inhibitors. Values calculated based upon yield data shown in **(B)**. Values in both **(B)** and **(C)** represent average ± SEM.

Previous studies have shown that Retro-1 and Retro-2 can inhibit the retrograde pathway and cause a reduction in EV particle formation during VACV and MPXV infection (Harrison et al., 2016; Sivan et al., 2016). To confirm that the decrease in viral spread observed earlier was attributable to a reduction in EV but not MV particle production, we infected cells with VACV IHDJ, a VACV strain that produces a relatively higher proportion of EV particles (Figure 3A). We observed that at 2.5 μM all three compounds significantly decreased EV yield compared to control (Figure 3B). While none of the three compounds significantly affected MV formation, a small reduction in MV yield was observed in case of PA104 (Figure 3B). We hypothesize that this reduction in MV production could be due to effects on viral entry (Figure 2B). The reduction in EV yield among the compounds tested ranged between 54 and 89% (Figure 3C).

### PA104 Strongly Suppresses Actin Tail Formation in VACV-Infected Cells

**FIGURE 4:**
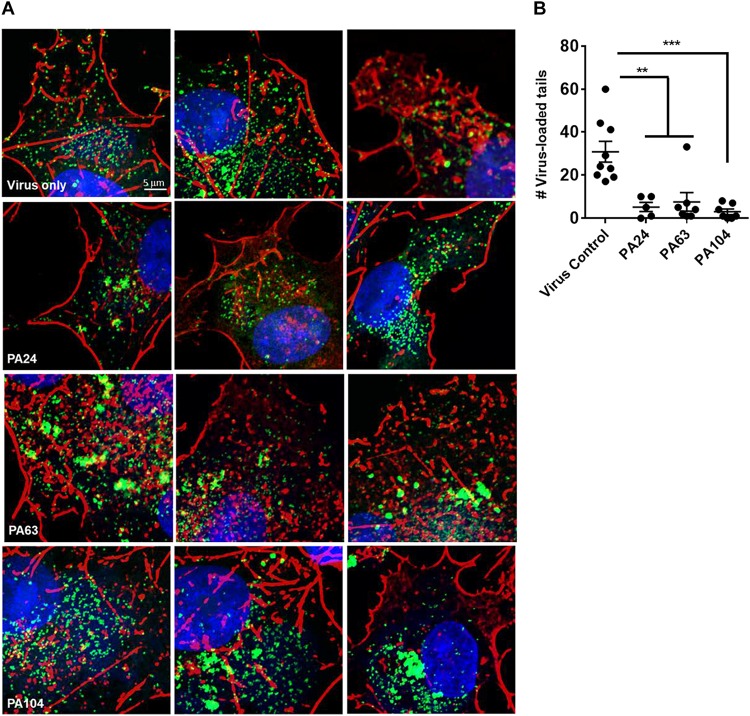
Inhibition of EV formation visualized by actin staining. **(A)** Confocal microscopy images of cells infected with VACV WR A4-YFP virus in the presence or absence of Retro-1 analogs PA63, PA24, and PA104. Few to no virus-loaded actin tails observed in the presence of Retro-1 analogs in comparison to virus-only treatment. Nuclei, actin, and VACV are stained blue, red, and green, respectively. Scale bars represent 5 μm. **(B)** Average number of virus-loaded actin tails per cell for each treatment. For each condition, virus-loaded actin tails from five to nine cells were counted (virus control: 9, PA24: 5, PA63: 9, and PA104: 7). Each dot represents one cell, and the average ± SEM for all four conditions are shown. Results of one-way analysis of variance and *post hoc* Tukey multiple-comparisons test comparing the four treatments are described: **p ≤ 0.005 and ***p ≤ 0.0005.

The double-membrane wrapping of MVs and subsequent formation of EVs in an infected cell triggers the polymerization of actin. This results in the formation of actin tails that can propel the EV particles to neighboring cells, causing cell-to-cell spread (Smith et al., 2002). Since we had observed a decrease in both viral spread and EV yield in the presence of Retro-1 analogs, we were interested in testing the effect of these compounds on the appearance of virus-loaded actin tails within cells. To this end, we infected cells with VACV in the presence or absence of PA24, PA63, and PA104 and visualized actin-associated virus particles using confocal microscopy. In the absence of the Retro-1 inhibitors, infected cells had several actin tails loaded with VACV at the tips (the viral particles appear as green dots due to the fusion of GFP with the core protein A4), which is characteristic of VACV infection (Figure 4 and Supplementary Figure S2). On the other hand, in all three inhibitor conditions, actin aggregates appeared shorter and did not resemble the clearly defined tails observed in the untreated control (Figure 4A). We enumerated the virus-loaded actin tails for each condition and found a significant decrease in virus-loaded tails in the presence of PA24, PA63, and PA104 (Figure 4B). In case of PA104, the reduction in actin tail staining was the most significant compared to the virus control. These data corroborate the results shown in Figures 3B,C, demonstrating that fewer virus-loaded actin tails appear in the presence of retrograde inhibitors, which results in a lower EV yield overall.

### PA104 Potently Inhibits the Spread of ST-246-Resistant Viruses

**FIGURE 5:**
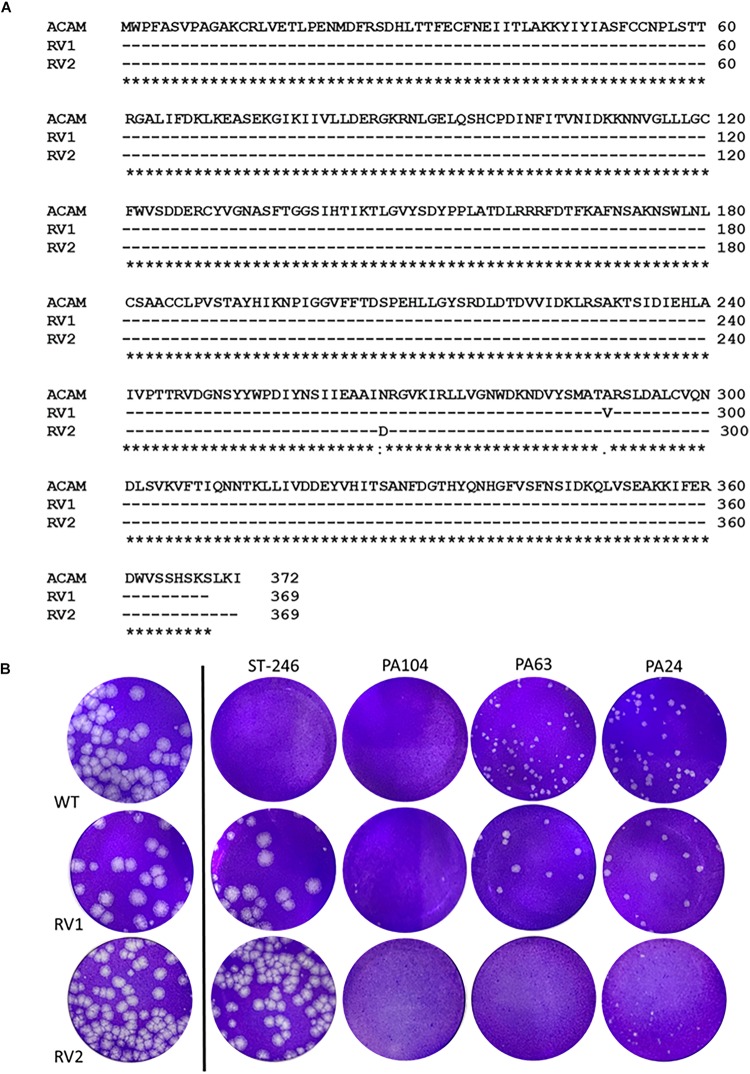
Retro-1 analogs PA104, PA24, and PA63 inhibit viral spread of ST-246-resistant VACV. **(A)** RV1 and RV2 sequence alignments showing preserved A290V and N267D mutations, respectively. Amino acid alignment of F13 of lab-grown RV1 viral isolates and RV2 compared to published ACAM strain (Lederman et al., 2012). **(B)** Plaque assay images showing observable decrease in viral plaque size of RV1 and RV2 virus in the presence of 10 μM PA104, PA24, and PA63.

ST-246, targets F13, a peripheral viral membrane protein, interfering with its intracellular localization (Yang et al., 2005). However, mutations in F13L gene can cause viral resistance to ST-246 as demonstrated previously (Yang et al., 2005; Lederman et al., 2012; Duraffour et al., 2015). To investigate whether the potent anti-VACV compounds identified in this study could inhibit ST-246–resistant virus, we infected cells with two different ST-246-resistant viruses in the presence of 10 μM PA104, PA24, and PA63. The two viruses used contained distinct resistance-conferring mutations; the first, hereby referred to as RV1, was isolated from the lesions of a PV patient by serial plaque purification in the presence of ST-246 (Lederman et al., 2012). We sequenced the F13L gene of our isolated RV1 and found the previously reported resistance mutation A290V (Figure 5A). The second virus, hereby referred to as RV2, was acquired from SIGA Technologies and contains the N267D resistance mutation (Smith et al., 2011).

While both the ST-246-resistant viruses formed large plaques in the presence or absence of 2 μM ST-246, a concentration of ST-246 over 50-fold higher than its IC50 value, treatment with PA104, PA24, and PA63 caused a considerable reduction in viral plaque size. As shown in Figure 5B, all three compounds substantially reduced the size of RV2 plaques. For RV1, on the other hand, PA104 was markedly more effective in reducing viral spread than PA24 and PA63 (Figure 5B). Overall, these data show that retrograde inhibitors can block EV formation and spread of ST-246-resistant VACV.

## Discussion

**FIGURE 6:**
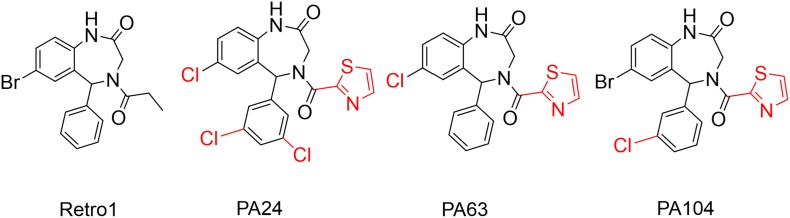
Structural modifications in PA104, PA24, and PA63. All modifications made to the original Retro-1 structure shown in red.

In this study, we describe the screening and characterization of a panel of compounds sharing the benzodiazepine scaffold of Retro-1. These efforts resulted in the discovery of three compounds, PA24, PA63, and PA104, with impressive antiviral activity against VACV, the most potent and promising of which is PA104. The structural differences between these analogs and their parent Retro-1 have been highlighted in Figure 6, and nuclear magnetic resonance (NMR) and mass spectrometry (MS) data are provided in Supplementary Data Sheets S1, S2. We demonstrated the following: (i) Retro-1 derivatives potently inhibit VACV spread *in vitro* with minimal cytotoxicity. (ii) The inhibitory effect was predominantly at a late stage of viral replication. (iii) EV formation is the main target of the analogs, which have little impact on MV yield. (iv) Retro-1 analogs can effectively decrease viral spread of ST-246-resistant viruses. In addition, we identified the Retro-1 derivative PA104 as a promising antiviral candidate with superior anti-VACV efficacy compared to the previously characterized Retro-1 and Retro-2, low cellular toxicity, and potent activity against ST-246-resistant viruses.

In total, 83 compounds were tested for their ability to decrease VACV spread *in vitro*. We observed that 17 of these compounds had comparable or more potent anti-VACV activity than Retro-1 and Retro-2 (IC_50_ and IC_90_ values ≤ Retro-1 and Retro-2). We focused on three compounds with high potency and low cytotoxicity, PA24, PA63, and PA104, and subsequently tested their impact on VACV entry and EV/MV yield. The most potent inhibitor of VACV spread, PA104, caused an observable reduction in luciferase activity even at 2.5 μM. In contrast, PA24 and PA63 only appeared to affect entry at higher concentrations. All three compounds tested minimally impacted MV yield while considerably reducing EV yield, except for PA104, which caused a small reduction in MV yield in addition to decreasing EV formation. The spread assay, entry assay, and MV/EV yield data together suggest that while the main mechanism of viral inhibition by Retro-1 derivatives is the inhibition of EV particle formation, select compounds such as PA104 may impact viral entry when present at concentrations greater than their effective inhibitory concentrations. These findings aligned well with previous characterizations of Retro-1 and Retro-2 activity *in vitro* (Harrison et al., 2016; Sivan et al., 2016).

Vaccinia virus replicates in the cytoplasm of the infected cells, resulting in the formation of infectious, single-membrane MV particles at the site of replication (Condit et al., 2006). Although infectious, these MV particles remain intracellular unless wrapped by two additional membranes of post-Golgi or endosomal origin to form EVs. Once the outer layer of the EV double membrane fuses with the plasma membrane, an EV can be propelled out of the infected cell by actin tails (Smith et al., 2002). Because viral spread and EV yield were greatly reduced in the presence of our panel of compounds, we visualized actin tail-associated EVs in infected cells using confocal microscopy. Fewer actin tails were detected in the presence of all three compounds tested, PA104, PA63, and PA24, with the greatest reduction observed in case of PA104.

We and others have previously reported the spontaneous development of ST-246 resistance, both *in vitro* (Yang et al., 2005; Duraffour et al., 2015) and clinically in a smallpox-vaccinated PV patient receiving ST-246 as part of their treatment regimen (Lederman et al., 2012). In healthy individuals, it is possible that the immune response might overcome such resistant viruses, if they arise. However, additional interventions may be required for at-risk groups that cannot mount a protective immune response, such as individuals with immune deficiencies. For such cases, it would be beneficial to have additional antiviral candidates that can be administered in combination with ST-246 and can suppress ST-246-resistant viruses. We tested the inhibitory potential of PA104, PA63, and PA24 against two different ST-246-resistant viruses and found that all three compounds reduced viral spread of both viruses, as determined by a reduction in viral plaque size. Interestingly, while RV2 was substantially inhibited by all three Retro-1 compounds, RV1 appeared to be less efficiently inhibited by PA24 and PA63. These differences in the levels of viral inhibition by the same compounds could potentially be caused by additional uncharacterized mutations in other genes, or strain differences between the viruses; whereas RV1 was an Acambis strain of VACV, RV2 was a Western Reserve strain. Nevertheless, PA104 treatment resulted in maximal inhibition of plaque size for wild-type as well as RV1 and RV2 ST-246-resistant viruses. We hypothesize that its superior capacity to inhibit these viruses compared to PA24 and PA63 could be due to its ability to target multiple steps of VACV infection, including viral entry and MV formation, in addition to EV formation (Figures 2, 3).

In all, given its high SI (>800), submicromolar effective concentration, and potent inhibition of ST-246-resistant viruses, PA104 emerges as a promising candidate for future *in vivo* exploration. Further, PA104 targets a host-specific process rather than a viral protein, offering two additional advantages. First, the absence of a direct viral target reduces the probability of viral resistance to the compound (Lin and Gallay, 2013; Kaufmann et al., 2018). Second is the possibility of broad-range applicability to other viruses and non-viral pathogens that utilize the retrograde pathway for entry or egress. We and others have previously shown that Retro-2 and its analogs can reduce *Leishmania*, polyomavirus, and papillomavirus infections, as well as inhibit Shiga toxin trafficking in cells (Noel et al., 2013; Carney et al., 2014; Craig et al., 2017). Recent reports have also investigated the effect of Retro-2 derivatives on HSV2 infections (Dai et al., 2018). A majority of these studies have focused primarily on Retro-2 derivatives, and at present, less is understood about the antipathogenic potential of Retro-1 and its derivatives. To our knowledge, this is the first study to provide an in-depth, large-scale characterization of the inhibitory potential of compounds structurally related to Retro-1. As the retrograde pathway plays an important role for several pathogens and toxins, further characterization of compounds such as PA104 and exploration of their *in vivo* activity could have important implications for the design of broad-spectrum therapeutics that have immense public health benefits. An in-depth structure–activity relationship study of PA104 and other structural analogs of Retro-1 is in progress and will be reported in due course.

## Data Availability Statement

The raw data supporting the conclusions of this article will be made available by the authors, without undue reservation, to any qualified researcher.

## Author Contributions

LP, JS, and PS contributed to the conception and design of the study. PA, AL, and JS designed and generated the benzodiazepine scaffold-containing compounds. LP and PS performed poxvirus experiments and wrote the first draft of the manuscript. AK and SS performed confocal imaging experiments. LP, PA, VO, JS, and PS edited the manuscript. All authors contributed to the manuscript revision and read and approved the submitted version.

## Conflict of Interest

The authors declare that the research was conducted in the absence of any commercial or financial relationships that could be construed as a potential conflict of interest.

## Supplementary Material

FIGURE S1 Validation of luciferase-based viral entry assay. **(A)** Minimal input luciferase activity after 5 min infection at RT. Luciferase activity in cells infected for 5 min at RT was significantly lower than infection for 2 h at 37°C. ^****^ indicates an unpaired *t*-test *p*-value < 0.0001. **(B)** PA104 does not impact luciferase enzyme activity. No difference observed in RLU values in the presence of three concentrations of PA104. Both graphs show mean values ± SEM.

FIGURE S2 Fewer virus-loaded actin tails in the presence of Retro-1 inhibitors. Confocal microscopy images of cells infected with VACV WR A4-YFP virus in the presence or absence of Retro-1 analogs PA63, PA24, and PA104. Lower magnification images of cells pictured in Figure 4. Scale bars represent 10 μm and white boxes indicate the area within each cell that was magnified to create Figure 4.

DATA SHEET S1 NMR and MS data for PA104, PA24, and PA63.
