# Supplementary figures and images for: Discovery of Retro-1 Analogs Exhibiting Enhanced Anti-vaccinia Virus Activity
Source: Front Microbiol. 2020 Apr 23;11:603. doi: 10.3389/fmicb.2020.00603 (PMC7190985; doi:10.3389/fmicb.2020.00603)

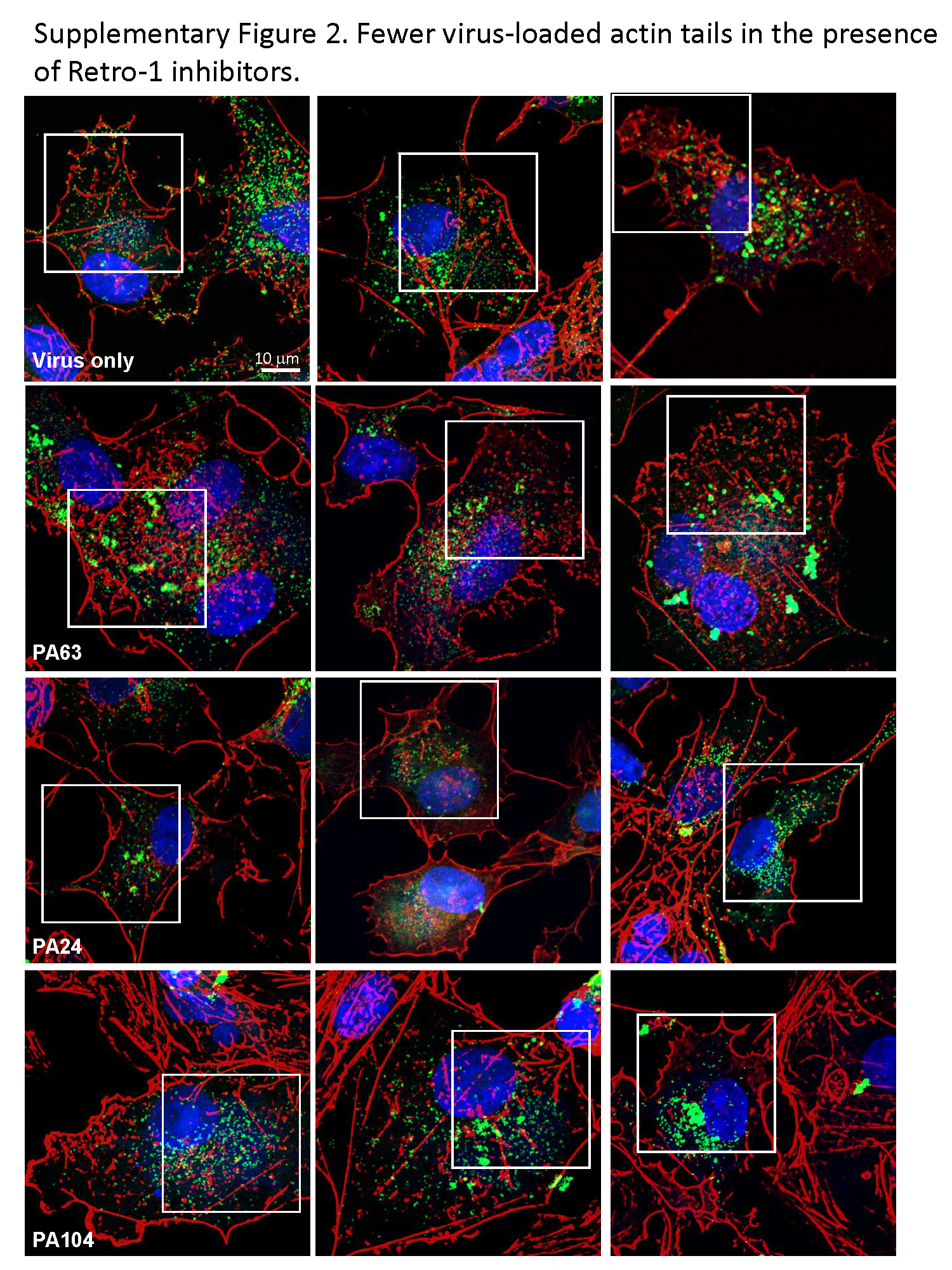

Supplement: FIGURE S2 — Fewer virus-loaded actin tails in the presence of Retro-1 inhibitors. Confocal microscopy images of cells infected with VACV WR A4-YFP virus in the presence or absence of Retro-1 analogs PA63, PA24, and PA104. Lower magnification images of cells pictured in Figure 4. Scale bars represent 10 μm and white boxes indicate the area within each cell that was magnified to create Figure 4. [file Image_2.TIFF]
