# Supplementary material for: Discovery of Retro-1 Analogs Exhibiting Enhanced Anti-vaccinia Virus Activity
Source: Front Microbiol. 2020 Apr 23;11:603. doi: 10.3389/fmicb.2020.00603 (PMC7190985; doi:10.3389/fmicb.2020.00603)
Supplement: DATA SHEET S1 — NMR and MS data for PA104, PA24, and PA63. [file Data_Sheet_1.PDF]

PA24, PA63 and PA104 are structurally related to Retro1, yet exhibit markedly enhanced potency as antivirals. A full disclosure of the origins of these compounds and their structure-activity relationships will be reported separately in due course.

Supporting Information

Characterization data of analogs

^{1}H and ^{13}C NMR spectra were recorded on a Bruker Avance 600 MHz spectrometer. Chemical shifts are (on a δ scale) relative to residual solvent $CDCl_3$ (7.27 ppm). All high-resolution mass spectra were recorded by Brown University staff using a Jeol JMS-600H spectrometer.

Characterization data for PA24 (7-chloro-5-(3,5-dichlorophenyl)-4-propionyl-1,3,4,5-tetrahydro-2H-benzo[e][1,4]diazepin-2-one)

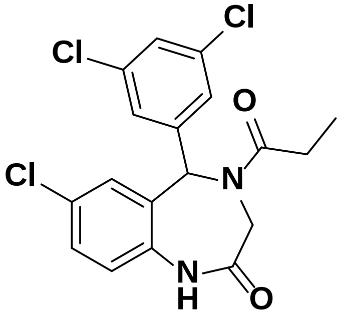

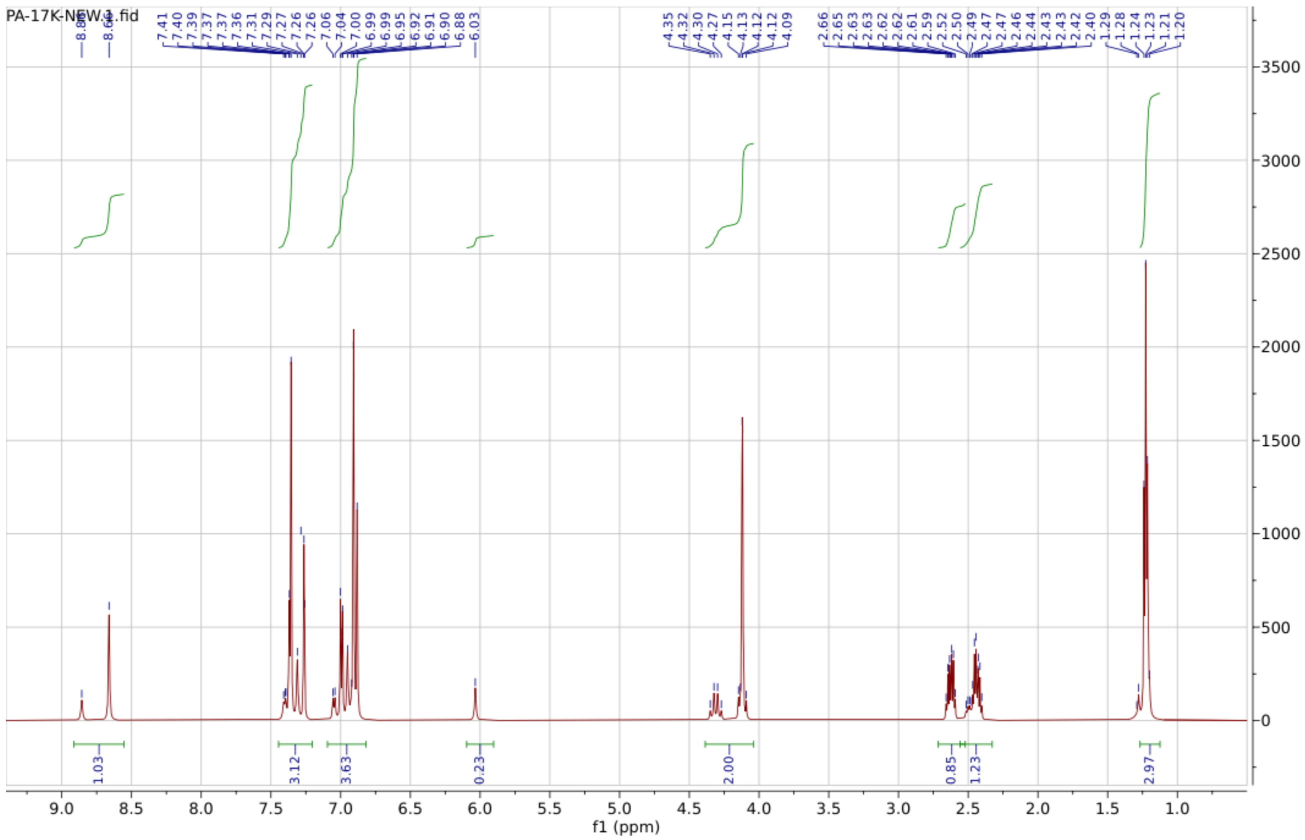

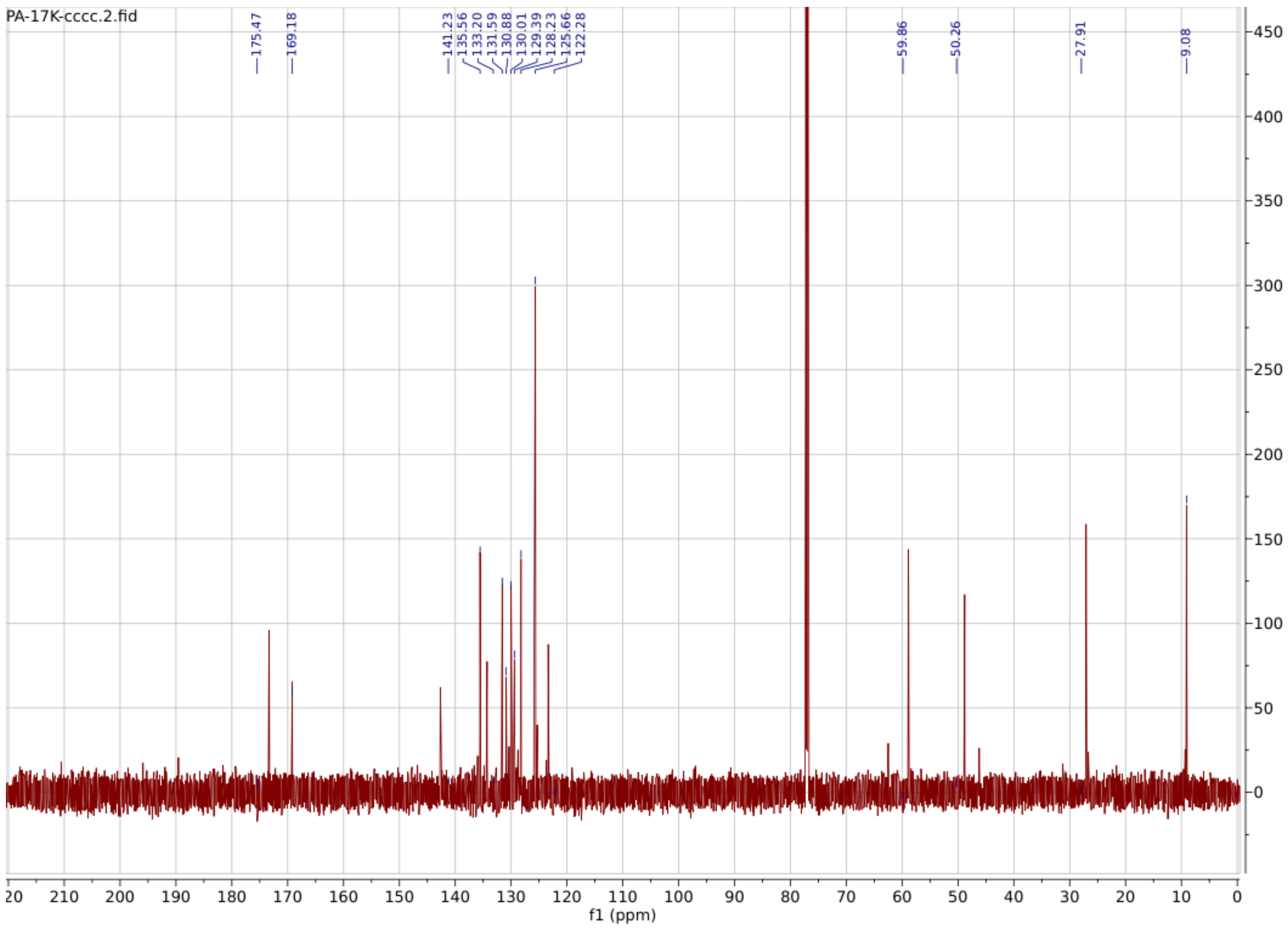

Characterization data for PA24 (7-chloro-5-(3,5-dichlorophenyl)-4-propionyl-1,3,4,5-tetrahydro-2H-benzo[e][1,4]diazepin-2-one)

¹H NMR (600 MHz, Chloroform-*d*) δ 8.76 (s, 1H), 7.47 – 7.13 (m, 3H), 7.08 – 6.65 (m, 4H), 6.03 (s, 0.3H), 4.42 – 3.88 (m, 2H), 2.84 – 2.20 (m, 2H), 1.23 (t, *J* = 7.4 Hz, 3H).

¹³C NMR (151 MHz, CDCl3) δ 175.47, 169.18, 141.23, 135.56, 133.20, 131.59, 130.88, 130.01, 129.39, 128.23, 125.66, 122.28, 59.86, 50.26, 27.91, 9.08.

HRMS (ESI): m/z calculated for $C_{18}H_{15}Cl_3N_2O_2$ $[M+H]^+$: 396.0199, found: 397.0268.

Characterization data for PA104 (7-bromo-5-(3-chlorophenyl)-4-(thiazole-2-carbonyl)-1,3,4,5-tetrahydro-2H-benzo[e][1,4]diazepin-2-one)

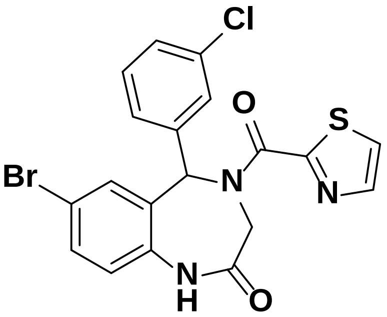

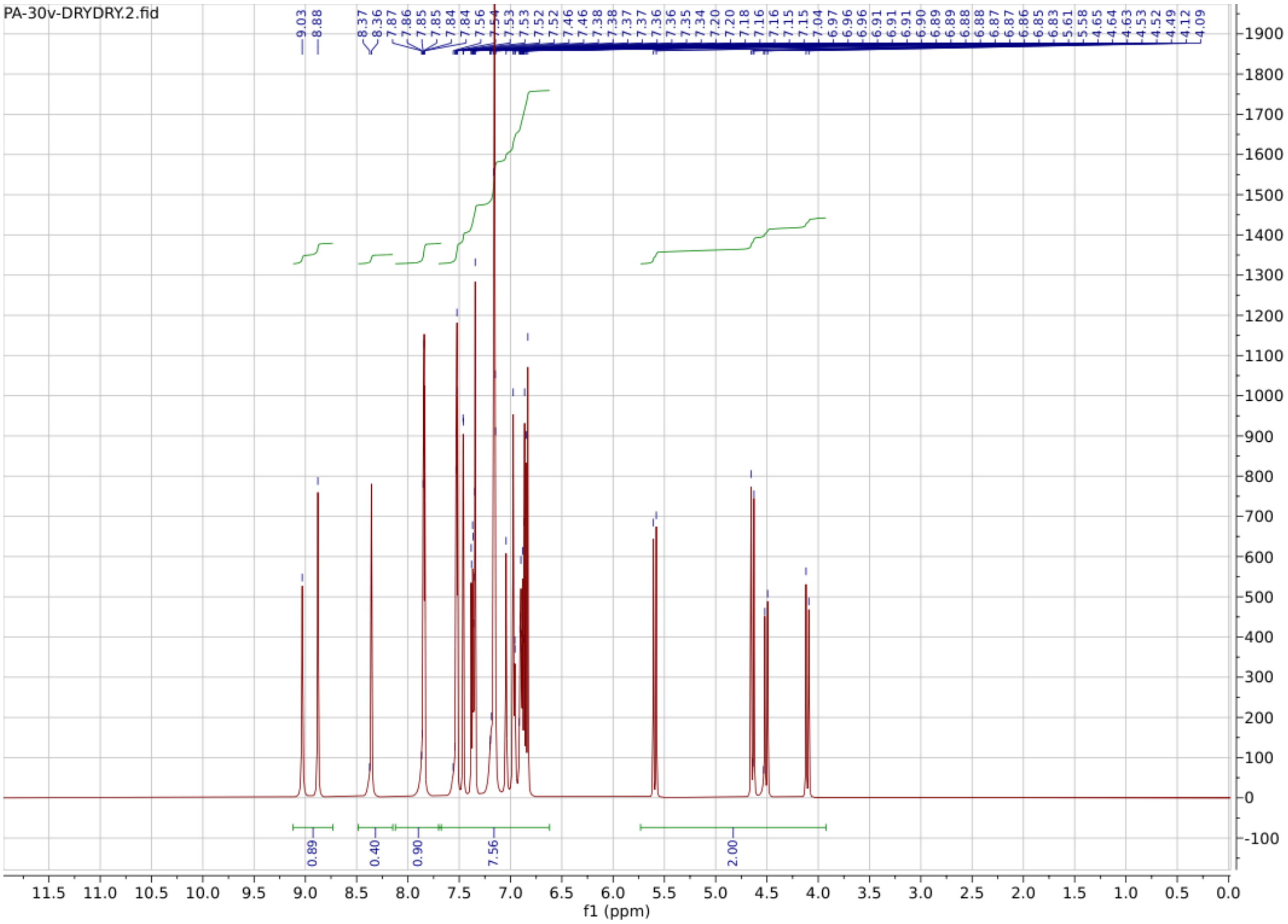

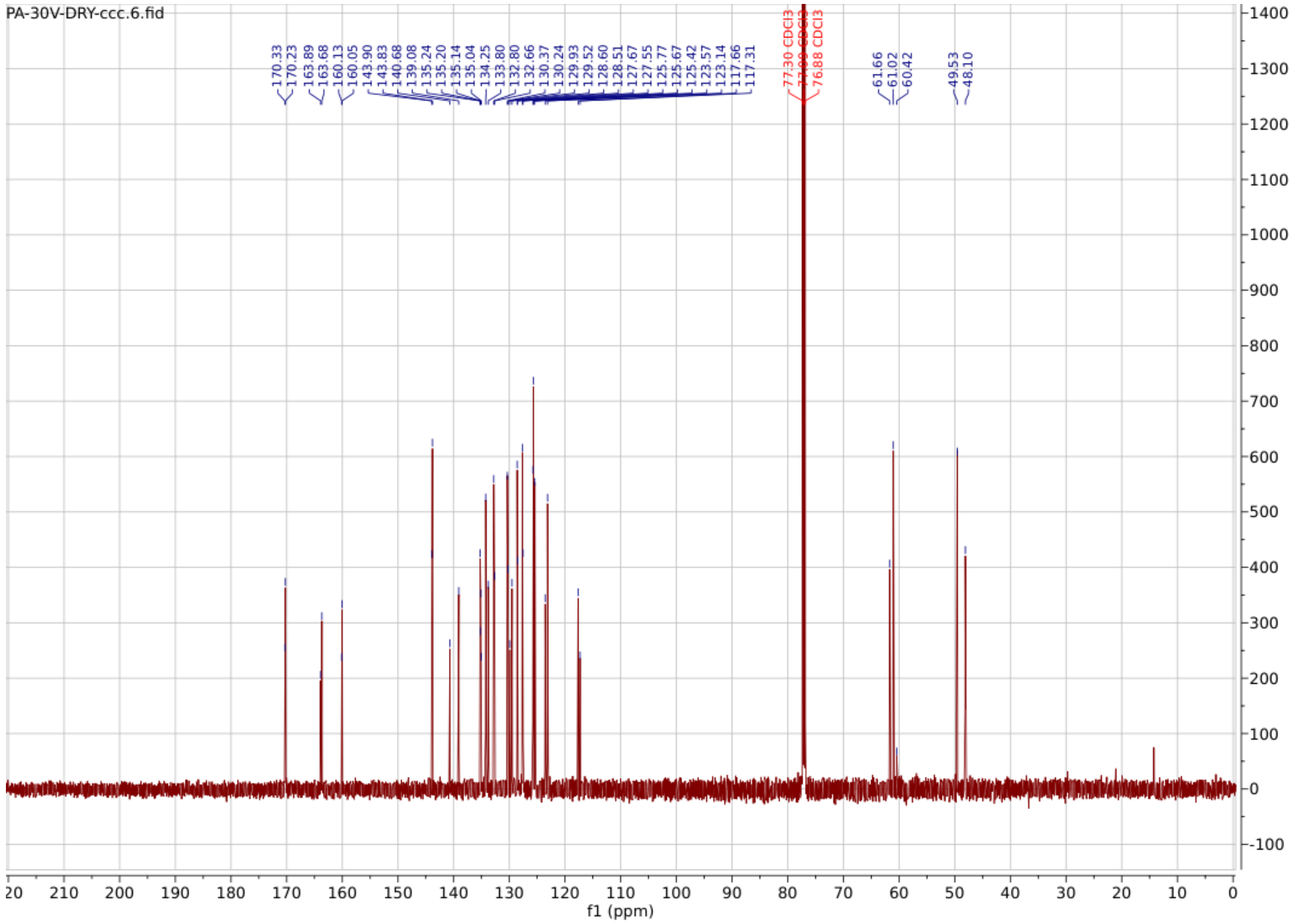

Characterization data for PA104 (7-bromo-5-(3-chlorophenyl)-4-(thiazole-2-carbonyl)-1,3,4,5-tetrahydro-2H-benzo[e][1,4]diazepin-2-one)

^{1}H NMR (600 MHz, Chloroform-*d*) δ 8.95 (d, *J* = 91.5 Hz, 1H), 7.84 (dd, *J* = 6.1, 3.2 Hz, 1H), 7.57 – 7.43 (m, 2H), 7.39 – 7.32 (m, 1H), 7.16 (t, *J* = 4.6 Hz, 2H), 7.07 – 6.93 (m, 1H), 6.93 – 6.81 (m, 2H), 5.59 – 4.12 (5.59 (d, *J* = 17.0 Hz), 4.64 (d, *J* = 17.1 Hz), 4.51(d, *J* = 16.5 Hz), 4.12 (d, *J* = 16.5 Hz), 2H).

^{13}C NMR (151 MHz, CDCl3) δ 170.33, 170.23, 163.89, 163.68, 160.13, 160.05, 143.90, 143.83, 140.68, 139.08, 135.24, 135.20, 135.14, 135.04, 134.25, 133.80, 132.80, 132.66, 130.37, 130.24, 129.93, 129.52, 128.60, 128.51, 127.67, 127.55, 125.77, 125.67, 125.42, 123.57, 123.14, 117.66, 117.31, 61.66, 61.02, 60.42, 49.53, 48.10. Peaks are duplicated because of the existence of rotamers

HRMS (ESI): m/z calculated for $C_{19}H_{13}BrClN_3O_2S$ $[M+H]^+$: 460.9600, found: 461.9668.

Characterization data for PA63 (7-chloro-5-phenyl-4-(thiazole-2-carbonyl)-1,3,4,5-tetrahydro-2H-benzo[e][1,4]diazepin-2-one)

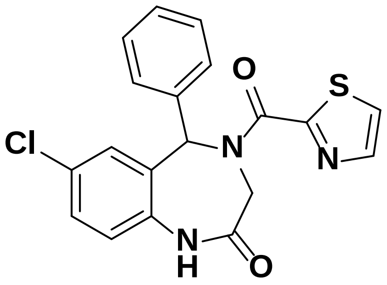

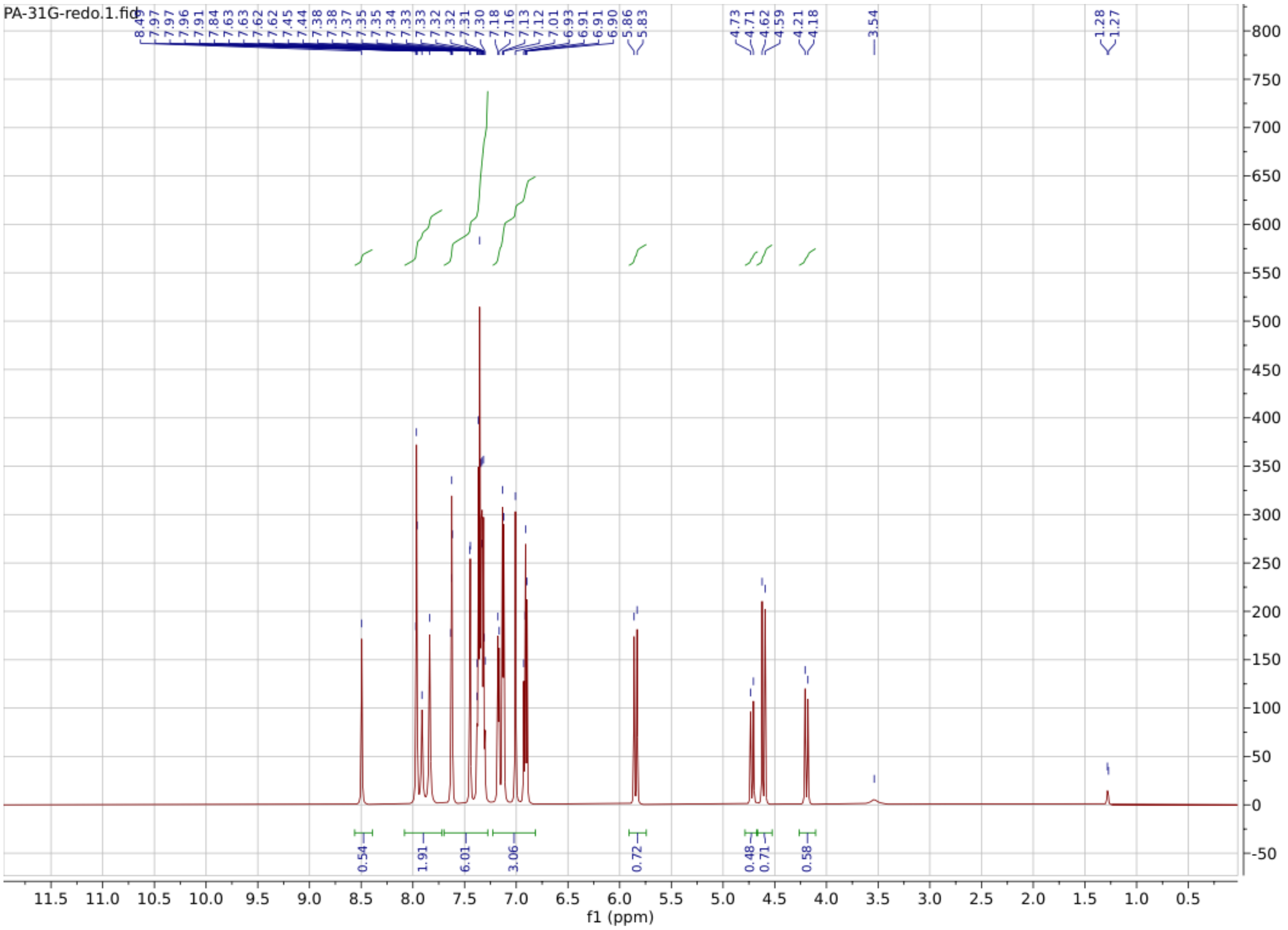

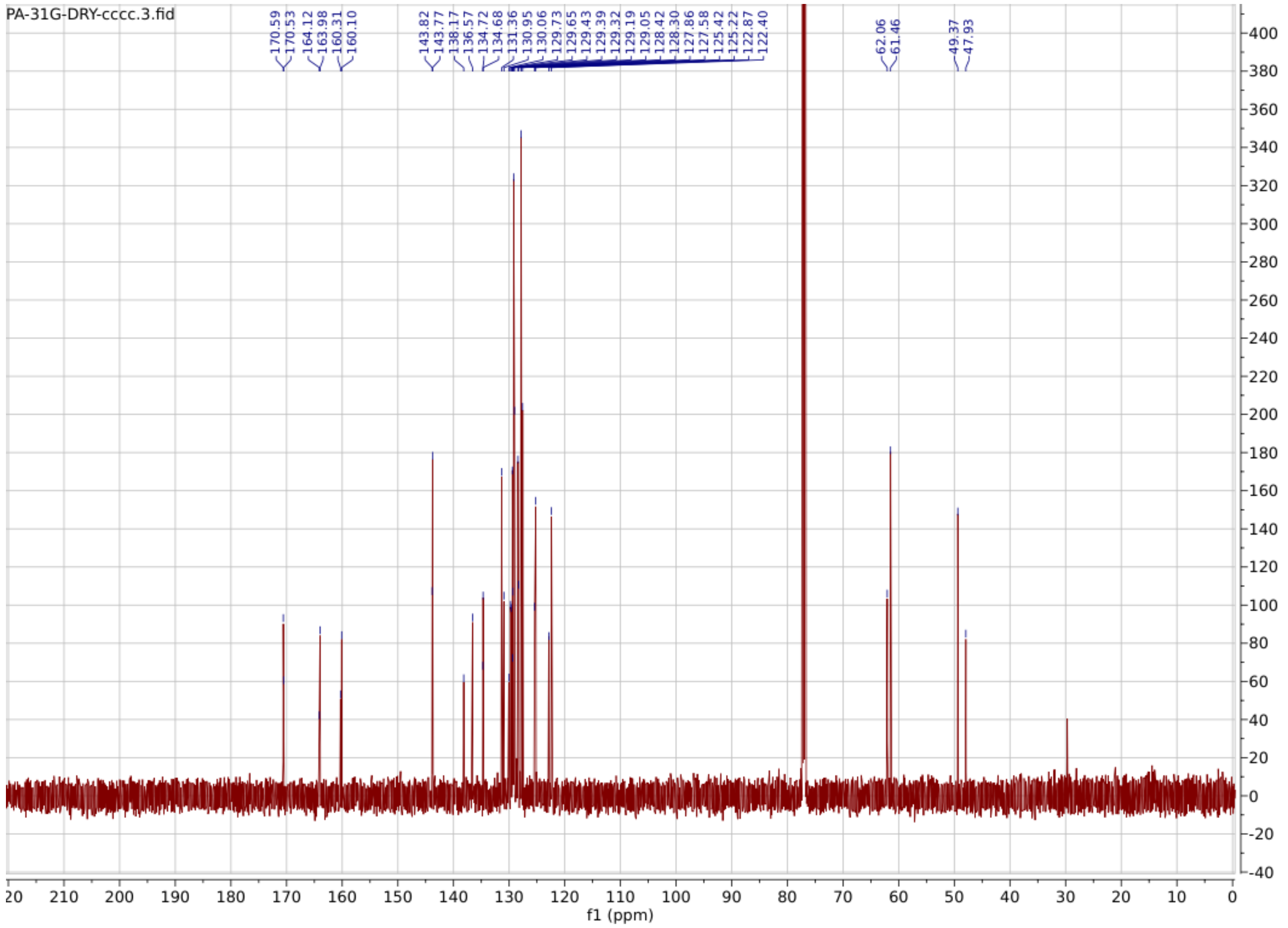

Characterization data for PA63 (7-chloro-5-phenyl-4-(thiazole-2-carbonyl)-1,3,4,5-tetrahydro-2H-benzo[e][1,4]diazepin-2-one)

^{1}H NMR (600 MHz, Chloroform-*d*) δ 8.49 (s, 1H), 8.08 – 7.72 (m, 2H), 7.70 – 7.28 (m, 6H), 7.23 – 6.82 (m, 3H), 5.85-4.19 (dd (4.70 (d, *J* = 16.7 Hz) 5.85 (d, *J* = 17.4 Hz), 4.61 (d, *J* = 17.4 Hz), 4.19 (d, *J* = 16.7 Hz), 2H).

^{13}C NMR (151 MHz, CDCl3) δ 170.59, 170.53, 164.12, 163.98, 160.31, 160.10, 143.82, 143.77, 138.17, 136.57, 134.72, 134.68, 131.36, 130.95, 130.06, 129.73, 129.65, 129.43, 129.39, 129.32, 129.19, 129.05, 128.42, 128.30, 127.86, 127.58, 125.42, 125.22, 122.87, 122.40, 62.06, 61.46, 49.37, 47.93. Peaks are duplicated because of the existence of rotamers

HRMS (ESI): m/z calculated for $C_{19}H_{14}ClN_3O_2S$ $[M+H]^+$: 383.0495, found: 384.0568.
