# Supplementary material for: Discovery of Retro-1 Analogs Exhibiting Enhanced Anti-vaccinia Virus Activity
Source: Front Microbiol. 2020 Apr 23;11:603. doi: 10.3389/fmicb.2020.00603 (PMC7190985; doi:10.3389/fmicb.2020.00603)
Supplement: Supplementary file 4 [file Data_Sheet_2.pdf]

SUPPLEMENTARY METHODS

Characterization data of analogs. 1H and 13C NMR spectra were recorded on a Bruker AVANCE 600 MHz spectrometer. Chemical shifts are (on a δ scale) relative to residual solvent CDCl3 (7.27 ppm). All high-resolution mass spectra were recorded by Brown University staff using a Jeol JMS-600H spectrometer.

Input luciferase detection. BSC40 cells were infected with VACV WR-Luc virus at MOI 3 either for 5 min at RT or for 2h at 37°C. Thereafter, cells were washed, and Reporter Lysis Buffer was added to the wells to lyse cells. After one freeze-thaw, luciferase activity was measured using the Luciferase Assay System (Promega, Madison, WI) according to manufacturer's instructions. Luciferase activity was measured using an ENSPIRE plate reader (PerkinElmer, Waltham, MA, United States). An unpaired t-test was performed to determine statistical significance using 16 replicate wells from each of the two treatment groups.

Impact of PA104 on luciferase activity. To test the effect of PA104 on luciferin activity, BSC40 cells were infected with VACV WR-Luc virus and lysed as described above. PA104 diluted in DMEM to three concentrations (20 µM, 10 µM and 5 µM) was added to lysed cells and mixed prior to measuring luciferase activity using the Luciferase Assay System. A total of 8 replicates were included for each treatment, including the virus only group.
